# Electroacupuncture Improves Ovarian Function in Rats With *Tripterygium* Glycoside-Induced Diminished Ovarian Reserve by Promoting the Polarization of M2 Macrophages and Inhibiting Inflammatory Responses

**DOI:** 10.1155/mi/1694470

**Published:** 2025-03-31

**Authors:** Jia Luo, Yantong Qin, Yaoyao Zhu, Yaoli Yin, Meihong Shen

**Affiliations:** ^1^School of Acupuncture-Moxibustion and Tuina and School of Health Preservation and Rehabilitation, Nanjing University of Chinese Medicine, Nanjing 210023, China; ^2^Department of Rehabilitation, Gaoyou Hospital of Traditional Chinese Medicine, Yangzhou 225600, China; ^3^Institute of Acupuncture and Moxibustion, China Academy of Chinese Medical Sciences, Beijing 100700, China; ^4^Key Laboratory of Acupuncture and Medicine Research of Ministry of Education, Nanjing University of Chinese Medicine, Nanjing 210023, China

## Abstract

Immunoinflammatory responses and macrophage polarisation are crucial for maintaining ovarian function. Moreover, electroacupuncture (EA) has been shown to protect ovarian function. However, the mechanisms by which EA improves ovarian function, including its effects on immunoinflammatory responses and macrophage polarisation, have not been determined. This study aimed to investigate the protective effects of EA on ovarian function in rats with diminished ovarian reserve (DOR) and to elucidate the regulatory mechanisms underlying inflammation and M1 and M2 macrophage polarisation. DOR models were established through the intragastric administration of 50 mg/kg *Tripterygium* glycoside suspension (TGs) for 14 consecutive days. The EA group received treatment at 2/100 Hz and 1.0 mA for 10 min using acupoints BL23, CV4 and CV12 for 14 days. Following the intervention, we employed various methodologies, including haematoxylin–eosin (H&E) staining, enzyme-linked immunosorbent assay (ELISA), flow cytometry, immunohistochemical (IHC) staining, western blotting and quantitative reverse transcriptase-polymerase chain reaction (PCR), to assess ovarian function, inflammatory factors and the expression levels of M1 and M2 macrophage-related factors. EA intervention reduced the oestrous cycle disorder rate in the rats compared with that in the DOR group, leading to an increase in growing follicles, a reduction in atretic follicles (AFs) and an enhancement of both the capillary (Cap) network and corpus luteum (CL) structure. This intervention also resulted in decreased serum levels of follicle-stimulating hormone (FSH), interferon-*γ* (IFN-*γ*) and tumour necrosis factor-*α* (TNF-*α*), along with increased levels of oestradiol (E_2_), interleukin-4 (IL-4) and interleukin-10 (IL-10). Furthermore, the number of M2 macrophages in the spleen increased, which was accompanied by elevated arginase 1 (Arg1) and decreased inducible nitric oxide synthase (iNOS) expression in the ovarian tissues. In summary, EA can restore the impaired ovarian function caused by TGs by promoting M2 macrophage polarisation and inhibiting inflammatory responses.

## 1. Introduction

Ovarian reserve function refers to the ability of follicles to grow and develop into fertilised follicles in the cortical area of the ovary, which reflects the reproductive potential of women [1]. Diminished ovarian reserve (DOR) is characterised by a decrease in the number and/or quality of ova in the ovaries of women of reproductive age, leading to abnormal secretion of reproductive hormones and a gradual decline in reproductive capacity [2]. Although the exact mechanism underlying DOR is not fully understood, numerous studies have suggested that various factors, such as age, surgery, radiation therapy, medication, underlying medical conditions and unhealthy lifestyle habits, contribute to its development [3]. Current treatment options for DOR typically involve assisted reproductive technology, ovulation induction and hormonal contraceptives, among others. While assisted reproductive technology can help address infertility in patients with DOR to some extent, it is associated with complications, such as ovarian hyperstimulation, low clinical pregnancy rates and ectopic pregnancies [4]. Although drug therapy is commonly employed to stimulate the growth of mature follicles (MFs) or regulate the menstrual cycle, it may also increase the risk of ovarian hyperstimulation [5]. Furthermore, these interventions may not effectively improve ovarian function and related clinical symptoms, potentially affecting the health of offspring [6, 7].

*Tripterygium* glycoside (TG), which is an immunosuppressant used for rheumatic and immune diseases, can lead to visceral toxicity, particularly reproductive toxicity [8] and immune-inflammatory responses by disrupting the immune function balance. DOR caused by this substance is often linked to immune inflammatory reactions, characterised by increased levels of interferon-*γ* (IFN-*γ*) and tumour necrosis factor-*α* (TNF-*α*), as well as decreased levels of interleukin-4 (IL-4) and interleukin-10 (IL-10) [9]. This results in local inflammatory infiltration of the ovarian tissues [10]. Inflammation and ovarian reserve function are closely related, as evidenced by the increased levels of inflammatory cytokines and the activation of pro-inflammatory signalling pathways in ageing ovaries with DOR [11]. Inhibition of inflammatory factors is associated with increased ovarian reserve and enhanced fertility [12]. Macrophages play a crucial role in regulating the innate immune system and responding to inflammation. They maintain tissue homeostasis by managing the initiation and resolution of inflammation and facilitating tissue repair following damage [13]. Macrophages are present in various tissues and exhibit heterogeneity and plasticity in response to changes in their microenvironment [14]; M1 and M2 macrophages are the primary types. Cells can transition between the M1 and M2 phenotypes during immune and inflammatory regulation [15, 16]. M1 macrophages primarily secrete pro-inflammatory molecules to combat invading pathogens, thereby, fulfilling a pro-inflammatory role. In contrast, M2 macrophages express a range of anti-inflammatory molecules that facilitate tissue regeneration and remodelling [17]. The pathogenesis of ovarian ageing may involve an imbalance between M1 and M2 macrophages [18]. M1 macrophage polarisation contributes to the onset of ovarian inflammation during ageing [19], whereas the extracellular vesicle (EV) of M2 macrophages can improve ovarian function and the inflammatory microenvironment of aged mice [18]. Nevertheless, the relationship between TG-induced DOR and macrophage polarisation remains unclear.

Acupuncture, including electroacupuncture (EA), is a safe and effective traditional Chinese medicine therapy widely used in the treatment of reproductive disorders [20]. It has been shown to regulate reproductive hormone levels in older women [21] and ovulation-inducing rats [22] and increase the number of growing follicles, thereby, improving ovarian reserve function. Scientific evidence supports the use of acupuncture to enhance ovarian reserve [23]. Research indicates that EA is effective in treating immune inflammatory reactions and autoimmune diseases [24, 25] and exerts an anti-inflammatory therapeutic effect by facilitating the shift of macrophages from the M1 to M2 type [26]. Our previous research has revealed that EA could regulate the immune balance and alleviate oxidative damage in DOR rats [27, 28]. However, it remains unclear whether the ability of EA to treat ovarian dysfunction in DOR rats is related to macrophage polarisation and inflammation. Therefore, this study aimed to investigate the mechanism underlying the protective effect of EA on ovarian function in a DOR rat model based on macrophage polarisation and inflammation. The results of this study provide an experimental foundation for the use of EA in clinical DOR treatment.

## 2. Materials and Methods

### 2.1. Animals and Ethics Statement

Beijing Vital River Laboratory Animal Technology Co., Ltd. provided 8-week-old female Sprague–Dawley (SD) rats (production licence number: SCXK (Beijing) 2021-0011), weighing 190 ± 10 g. The animals were reared at Nanjing University of Chinese Medicine's Experimental Animal Centre under conventional conditions (25 ± 2°C, 55% ± 5% humidity and 12-h light/dark cycle) with humane care. The disposal of animals during the experiment was in accordance with the guiding principles of the China Ethics Committee on Animal Research and the relevant provisions of the guiding opinions on being kind to experimental animals and the study was approved by the Animal Ethics Committee of Nanjing University of Chinese Medicine (Ethics No.: 202202A037).

### 2.2. Establishment of the DOR Model and Experimental Grouping

After 3 days of adaptive feeding and 10 days of oestrous cycle screening, 27 female SD rats with normal oestrous cycles were randomly divided into control, DOR, and EA groups, using a random number table method, with nine rats in each group. *Tripterygium* glycoside suspension (TGs) (SFDA approval number: Z43020138; Hunan Qianjin Xieli Pharmaceutical Co., Ltd., Zhuzhou, China) was used to build the DOR model in accordance with our earlier work [29]. Rats in the DOR and EA groups were gavaged with TGs at 50 mg/kg/day for 14 days. The control group received the same volume of physiological saline via daily oral gavage.

### 2.3. Intervention Methods

**Figure 1:**
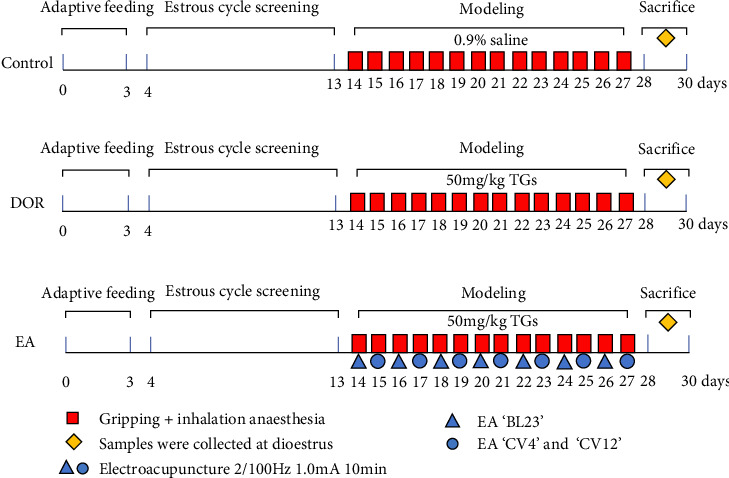
Experimental protocol.

After intragastric TGs delivery for 1 h, the rats in the EA group underwent EA. In accordance with the commonly used acupoint names and positioning in laboratory animals [30], the acupoints 'Shenshu' (BL23), 'Guanyuan' (CV4) and 'Zhongwan' (CV12) were chosen. On both sides of the spine, BL23 is situated 6 mm lateral to the second lumbar vertebra. The junction of the anterior midline and fourth nipple is the location of the rat umbilicus. CV4 is 25 mm below the umbilicus, and CV12 is 20 mm above it. Acupuncture was performed on both sides of 'BL23' acupoints on 1 day and 'CV4' and 'CV12' acupoints on another day. During the intervention, the acupoint area was shaved. After anaesthetising the rats with isoflurane (R510-22-10; Ruiwode Life Technology Co., Ltd., Shenzhen, China), disposable acupuncture needles (0.20 mm × 13 mm; Suzhou Medical Appliance Factory Co., Ltd., Suzhou, China) were used to puncture designated acupoints followed by the EA procedure. The abdominal acupoints were punctured 2 mm downward, whereas the dorsal acupoints were punctured 5 mm inward and downward. Moistened cotton balls, which served as irrelevant electrodes, were placed on the exterior of the bilateral 'BL23' points and connected to the positive terminal of the EA instrument. Meanwhile, the bilateral 'BL23' points were individually linked to the negative terminal. During the EA treatment of the abdominal acupoints, 'CV12' was connected to the positive pole and 'CV4' to the negative pole. The EA instrument (Jisheng Medical Co., Ltd., Leshan, China) was used with the following settings: a dense-sparse wave at a frequency of 2/100 Hz, current intensity of 1.0 mA, lasting for 10 min, administered once daily for 14 consecutive days. The intensity caused slight twitches in the local skin muscle. Model preparation and EA intervention began on the same day and lasted for 14 days. Both the DOR and control groups received fixed grip and inhalation anaesthesia at the same time each day. Following the intervention, dioestrus rats were injected intraperitoneally with 2% pentobarbital sodium (0.2 mL/100 g) to induce anaesthesia, obtain blood from the abdominal aorta and retrieve ovaries and spleens for use in later studies. The experimental protocol is illustrated in Figure 1.

### 2.4. Oestrous Cycle Assessment

At 8 am every morning, exfoliated cells from the rat vagina were collected using a cotton swab dipped in normal saline and subsequently smeared onto glass slides. Changes in the oestrous cycle of the rats were observed and recorded under a microscope. The oestrous cycle can typically be divided into four stages: pro-oestrus (P), oestrus (E), metoestrus (M) and dioestrus (D). Under the microscope, during the pro-oestrus stage, a significant number of round nucleated epithelial cells with distinct nuclei are typically observed. In the oestrus period, the field of view is dominated by anucleated cornified cells. The metoestrus phase is characterised by the simultaneous presence of anucleated cornified cells, nucleated epithelial cells and leukocytes, whereas the dioestrus stage is dominated by leukocytes [31]. An oestrous cycle disorder was identified if the smear revealed a significant extension of the cycle (≥6 days), stagnation at a certain stage for ≥3 days or if the cycle did not follow the normal order [32].

### 2.5. Haematoxylin–Eosin (H&E) Staining and Follicle Counting

The ovaries were fixed with paraformaldehyde (BL539A; Biosharp, Hefei, China) for 48 h, dehydrated, embedded and sliced (6 µm thick). After dewaxing and hydration, paraffin sections were stained with H&E (BL700B; Biosharp) for 3 min each, followed by sealing with neutral gum. The development of follicles, corpus luteum (CL) and granulosa cells in the rat ovarian tissues was observed under a 200x optical microscope (U-ND2-38; Olympus, Tokyo, Japan). Six left ovaries were randomly selected from each group, and six sections were chosen from each sample for follicle counting with a 10-section interval between each section. The counting method has been previously described in detail [10].

### 2.6. Enzyme-Linked Immunosorbent Assay (ELISA)

The levels of reproductive hormones (follicle-stimulating hormone (FSH) and oestradiol (E_2_)) and inflammatory cytokines (IFN-*γ*, TNF-*α*, IL-4 and IL-10) were measured by ELISA (cat. no. E-EL-R0391c, E-EL-0152c, E-EL-R0009c, E-EL-R2856c, E-EL-R0014c and E-EL-R0016c; Elabscience, Wuhan, China), following strict operational instructions.

### 2.7. Flow Cytometry

Fresh rat spleens were cut, ground, rinsed with phosphate-buffered saline (PBS) and passed through a 150-mesh sieve. The resulting mixture was centrifuged at 350 × *g* for 5 min at 4°C. After discarding the supernatant, the cells were lysed using red blood cell lysis buffer, followed by another round of centrifugation and removal of the supernatant. The cells were resuspended in PBS and incubated with F4/80 (sc-377009 FITC; Santa Cruz Biotechnology, Dallas, TX, USA), CD11b (201818; BioLegend, San Diego, CA, USA) and CD86 (200307; BioLegend) antibodies. After incubation, Cyto-Fast Perm Wash solution (426803; BioLegend) was used to break the membrane, followed by the addition of CD206 antibody (sc-58986 AF647; Santa Cruz Biotechnology). The cells were resuspended in PBS after centrifugation. Flow cytometry (Gallios; Beckman, Brea, CA, USA) was then employed to detect the macrophage surface markers F4/80 and CD11b, as well as the expression of CD86 and CD206 in M1 and M2 macrophages. FlowJo software was used to analyse the proportions of M1 and M2 macrophages and the ratio of M1 to M2 macrophages.

### 2.8. Quantitative Reverse Transcription-Polymerase Chain Reaction (qRT-PCR)

qRT-PCR was used to measure the mRNA expression of *iNOS* and *Arg1*. Ovarian tissues were ground, and RNA was extracted using TRIzol. RT was then performed using 5 μg RNA to obtain cDNA, followed by real-time PCR. PCR kits (11141ES60 and 11202ES08) were purchased from Yeasen Biotechnology Co., Ltd. (Shanghai, China), and primers were synthesised by Generay Biotechnology Co., Ltd. (Shanghai, China). The primer sequences used are listed in Table 1.

### 2.9. Immunohistochemical (IHC) Staining

Paraffin sections were dewaxed, antigens were repaired with sodium citrate and sections were incubated with iNOS (YT3169; ImmunoWay Biotechnology, Plano, TX, USA) or Arg1 (16001-1-AP; Proteintech, Wuhan, China) primary antibodies (both diluted, 1:800) overnight at 4°C. The following day, the sections were incubated sequentially with the secondary antibodies and strept–avidin biotin complex. Diaminobenzidine (DAB) was added under low light conditions to facilitate colour development. The IHC (SA1022) and DAB chromogenic kits (AR1022) were purchased from Boster Biological Technology Co., Ltd. (Wuhan, China). After colour development, the output was rinsed with tap and distilled water for 10 min. Subsequently, haematoxylin restaining, alcohol colour separation and dehydration were performed, followed by xylene transparency, neutral gum sealing and microscopic observation (U-ND6-2; Olympus) at 400x magnification. The average optical density in the visual field was determined using the ImageJ software.

### 2.10. Western Blotting

Proteins were extracted from frozen ovaries by adding RIPA lysis buffer (FD009; Fude Biological Technology Co., Ltd., Changsha, China) and a protease inhibitor, followed by grinding and homogenisation. The protein concentration was measured using the bicinchoninic acid assay method (BL521A; Biosharp), and the remaining protein was denatured in a water bath at 95°C for 10 min. The gels were prepared using an SDS-PAGE Gel Preparation Kit (P0012A; Beyotime, Shanghai, China). After sampling, electrophoresis and film transfer, the membrane was sealed with 5% skim milk for 2 h, and the primary antibody (iNOS 1:1000, Arg1 1:1000 and tubulin (FD0064, Fude Biological Technology Co., Ltd.) 1:5000) was added and incubated overnight at 4°C. Subsequent steps included washing the membrane, incubation with secondary antibodies (sheep anti-rabbit 1:10,000 and sheep anti-mouse 1:10,000), exposure to enhanced chemiluminescence hypersensitive chemiluminescence solution (FD8020; Fude Biological Technology Co., Ltd.) for imaging and quantitative analysis of the grey values of the protein bands using ImageJ software.

### 2.11. Statistical Analysis

The experimental data were analysed using SPSS software (version 26.0; IBM, Armonk, NY, USA). Normally distributed data are presented as mean ± standard deviation. Group comparisons were conducted using one-way analysis of variance. When homogeneity of variance was satisfied in pairwise comparisons, the least significant difference method was employed. Conversely, when homogeneity of variance was not met, the Tamhane method was utilised. The incidences of oestrous cycle disorders were compared using the Fisher's exact test. The level of statistical significance was set at *p* < 0.05.

## 3. Results

### 3.1. EA Improved Ovarian Dysfunction Induced by TGs

**Figure 2:**
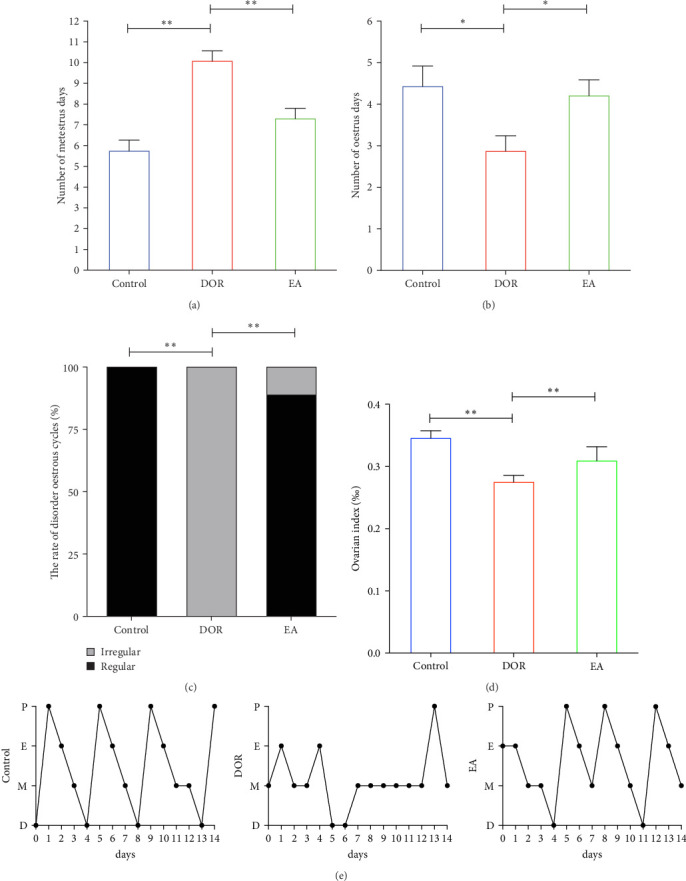
Impact of electroacupuncture (EA) on the oestrous cycle and ovarian index in rats with diminished ovarian reserve (DOR). Various parameters were evaluated, including (A) the number of metoestrus days (*n* = 9), (B) the number of oestrus days (*n* = 9), (C) a percentage of oestrous cycle disorders (*n* = 9), (D) ovarian index (*n* = 6) and (E) typical images of the oestrous cycle in each group. The results are presented as the mean ± standard deviation. Statistical significance is denoted as *⁣*^*∗*^*p* < 0.05 and *⁣*^*∗∗*^*p* < 0.01.

As shown in Figure 2A,B, compared with the estimates in the control group, rats in the DOR group exhibited an increased number of metoestrus days (*p* < 0.01) and a decreased number of oestrus days (*p* < 0.05). They also experienced completely irregular oestrous cycles (*p* < 0.01; Figure 2C). Following the EA intervention, the oestrous cycle disorder in the DOR model rats was rectified (*p* < 0.01), resulting in a reduction in metoestrus days (*p* < 0.01), an increase in oestrus days (*p* < 0.05) and a decrease in the rate of oestrous cycle irregularity. Furthermore, as illustrated in Figure 2D, intragastric administration of TGs reduced the ovarian index (%; (bilateral ovarian wet weight (mg)/rat body weight (g)) × 1000) in rats (*p* < 0.01), which was subsequently increased after EA intervention (*p* < 0.01). Typical images of the oestrous cycle in each group are shown in Figure 2E.

**Figure 3:**
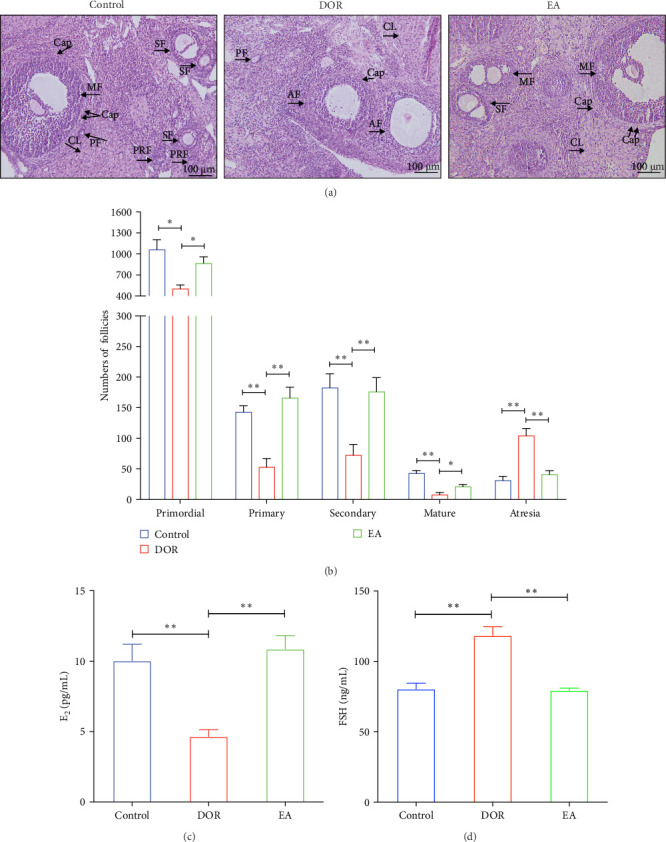
Effects of electroacupuncture (EA) on ovarian histomorphology, follicle count and serum reproductive hormone levels in rats with diminished ovarian reserve (DOR). (A) Representative haematoxylin–eosin (H&E) staining images of the ovarian tissues. AF, atretic follicle; Cap, capillary; CL, corpus luteum; MF, mature follicle; PF, primary follicle; PRF, primordial follicle; SF, secondary follicle. (B) Follicle counts at all levels in the ovarian sections (*n* = 6). (C, D) Serum levels of oestradiol (E_2_; *n* = 7) and follicle-stimulating hormone (FSH; *n* = 9). The results are presented as the mean ± standard deviation. Statistical significance is denoted as *⁣*^*∗*^*p* < 0.05 and *⁣*^*∗∗*^*p* < 0.01.

Histomorphological observations of rat ovaries revealed distinct differences among the control, DOR and EA groups (Figure 3A). In the control group, healthy follicles at various developmental stages were visible under a light microscope, with antral follicles displaying fullness and well-organised layers of granulosa cells, whereas atretic follicles (AFs) were rare. The structure of the CL was dense, with an abundance of capillaries surrounding the follicles. Conversely, the DOR group exhibited a significant decrease in the number of follicles across all stages of development (Figure 3B), with primordial, primary, secondary and MFs all affected (*p* < 0.05, *p* < 0.01, *p* < 0.01 and *p* < 0.01 compared with the control group, respectively). Additionally, the granulosa cell layer appeared diminished and loosely arranged with an increase in AFs (*p* < 0.01). The structure of the CL was loose, with a limited distribution of capillaries around the follicles. Subsequent EA treatment resulted in the restoration of follicle numbers (*p* < 0.05, *p* < 0.01, *p* < 0.01 and *p* < 0.05, respectively) and a reduction in AFs (*p* < 0.01). The structure of the CL in the EA group was relatively dense, and there was a widespread distribution of capillaries surrounding the follicles, which contributed to the improvement of ovarian morphology.

The pathological manifestations of DOR are characterised by hormonal imbalances, specifically marked by elevated FSH and decreased E_2_ levels. In our study, the DOR group exhibited significantly lower serum E_2_ levels (*p* < 0.01) and higher FSH levels (*p* < 0.01) than the control group. In contrast, the EA group exhibited significantly higher E_2_ levels (*p* < 0.01) and lower FSH levels (*p* < 0.01) than the DOR group (Figure 3C,D).

### 3.2. EA Reduced the Levels of Inflammatory Cytokines in DOR Rats

**Figure 4:**
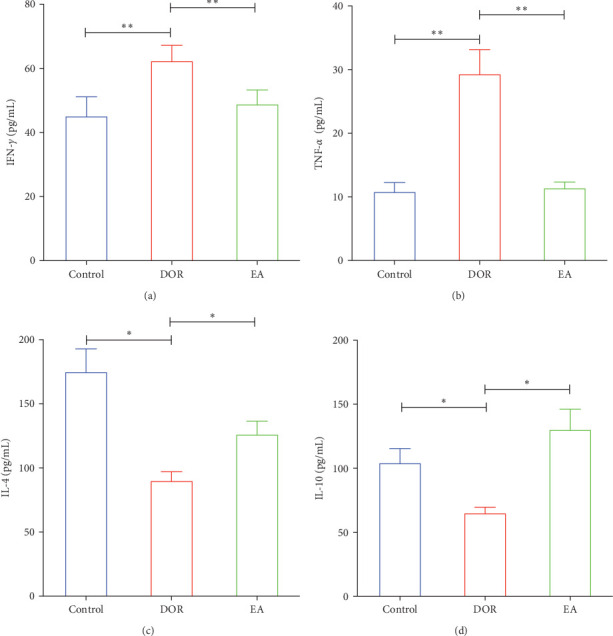
Effects of electroacupuncture (EA) treatment on inflammation. (A) Serum interferon-*γ* (IFN-*γ*) level (*n* = 8). (B) Serum tumour necrosis factor-*α* (TNF-*α*) level (*n* = 9). (C) Serum interleukin-4 (IL-4) level (*n* = 6). (D) Serum interleukin-10 (IL-10) level (*n* = 9). The results are presented as the mean ± standard deviation. Statistical significance is denoted as *⁣*^*∗*^*p* < 0.05 and *⁣*^*∗∗*^*p* < 0.01.

Previous findings have indicated that DOR rats show inflammatory reactions involving pro-inflammatory cytokines and anti-inflammatory cytokines, which play crucial roles in macrophage polarisation and immune response regulation. In this study, as shown in Figure 4A–D, the DOR group displayed elevated levels of IFN-*γ* (*p* < 0.01) and TNF-*α* (*p* < 0.01) along with decreased IL-4 (*p* < 0.05) and IL-10 (*P* < 0.05) levels in the serum compared with levels in the control group. Following treatment with EA, significant decreases in IFN-*γ* (*p* < 0.01) and TNF-*α* levels (*p* < 0.01) coupled with increases in IL-4 (*p* < 0.05) and IL-10 levels (*p* < 0.05) were observed in the EA group when compared with the DOR group.

### 3.3. EA Promoted the Polarisation of M2 Macrophages and Regulated the Balance of M1/M2 Macrophages

**Figure 5:**
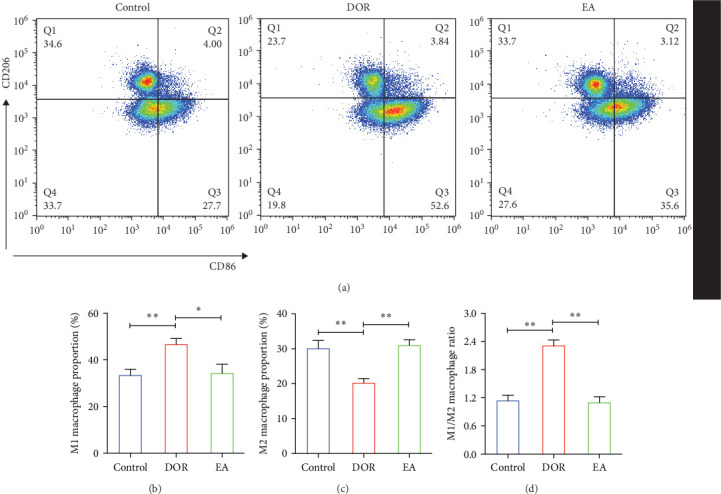
Effect of electroacupuncture (EA) on macrophage polarisation. (A) Flow cytometric analysis of M1 and M2 macrophage expression in rat spleen cells. (M1 macrophages: CD86^+^CD206^−^ region; M2 macrophages: CD86^−^CD206^+^ region). (B) The proportion of M1 macrophages (*n* = 5). (C) The proportion of M2 macrophages (*n* = 5). (D) The ratio of M1/M2 macrophages (*n* = 5). The results are presented as the mean ± standard deviation. Statistical significance is denoted as *⁣*^*∗*^*p* < 0.05 and *⁣*^*∗∗*^*p* < 0.01.

Macrophages exhibit different polarisation states, with M1 being classically activated and M2 being alternatively activated. In this study, as illustrated in Figure 5A–D, flow cytometry analyses of the spleen in the DOR group revealed that the cells were concentrated in the CD86^+^CD206^−^ region, revealing a significantly higher proportion of M1 macrophages (*p* < 0.01) and M1/M2 macrophage ratio (*p* < 0.01) than those in the control group. In contrast, the EA group exhibited a distinct shift, characterised by the dispersion of cells in the CD86^+^CD206^−^ region, along with an increase in the population of cells within the CD86^−^CD206^+^ region. This suggested a significant increase in the proportion of M2 macrophages (*p* < 0.01) and a decrease in the M1/M2 macrophage ratio (*p* < 0.01).

**Figure 6:**
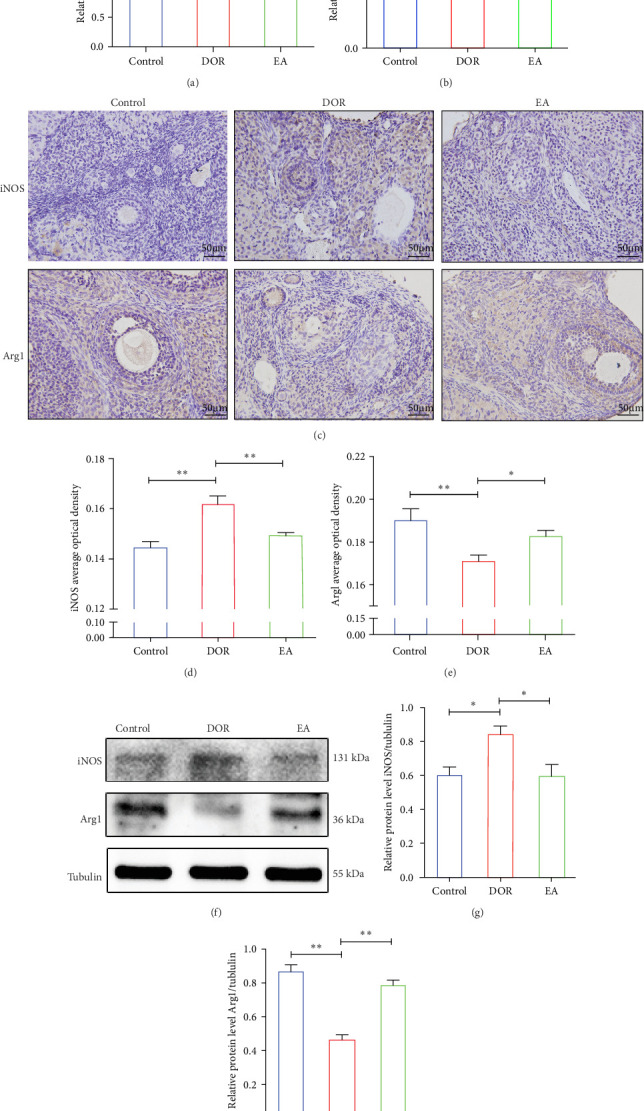
Effect of electroacupuncture (EA) on iNOS and Arg1 expression. (A, B) Relative expression levels of *iNOS* and *Arg1* mRNA in rat ovarian tissues (*n* = 5). (C) Immunohistochemical (IHC) analysis of rat ovarian sections. (D, E) The IHC average optical density (*n* = 6). (F–H) iNOS and Arg1 protein levels (*n* = 4). The results are presented as the mean ± standard deviation. Statistical significance is denoted as *⁣*^*∗*^*p* < 0.05 and *⁣*^*∗∗*^*p* < 0.01.

Moreover, macrophages exhibit antagonistic arginine metabolic pathways, with M1 macrophages characterised by iNOS expression, whereas M2 macrophages are characterised by the expression of arginase 1 (Arg1). In this study, the ovaries of DOR rats showed significantly higher relative expression levels of *iNOS* mRNA (*p* < 0.01) and lower relative expression levels of *Arg1* mRNA (*p* < 0.05) than those in the control group (Figure 6A,B). Rats in the EA group exhibited lower relative expression levels of *iNOS* mRNA (*p* < 0.01) in their ovaries than those in the DOR group, while exhibiting a significant increase in the relative expression of *Arg1* mRNA (*p* < 0.05).

The IHC results, shown in Figure 6C–E, revealed that in the DOR group, there was a notable augmentation in iNOS-positive cell expression and average optical density (*p* < 0.01), accompanied by lower levels of Arg1-positive cell expression and average optical density (*p* < 0.01) compared with those in the control group. Conversely, the EA group exhibited a decline in both iNOS-positive cells and average optical density (*P* < 0.01), along with a marked increase in Arg1-positive cell expression (*P* < 0.05) compared with the DOR group.

In line with the IHC findings, western blotting results displayed a consistent pattern (Figure 6F–H). Compared with the control group, the DOR group demonstrated an increase in iNOS protein expression (*p* < 0.05), whereas Arg1 expression decreased substantially (*p* < 0.01). Following EA intervention, a significant decrease in iNOS protein expression (*p* < 0.05) and a significant increase in Arg1 protein expression (*p* < 0.01) were observed, consistent with the previously mentioned trends.

## 4. Discussion

In this study, we utilised TGs to establish a DOR model for analysis of the ovarian protective mechanisms of EA, with a focus on the roles of macrophage polarisation and inflammation regulation. Our research demonstrates that EA can mitigate the decline in ovarian reserve function, as evidenced by the improvement in oestrous cycle regularity and ovarian tissue morphology and the restoration of disrupted reproductive hormone levels. The preservation of ovarian function may involve the promotion of macrophage polarisation towards the M2 phenotype and inhibition of inflammatory responses. These findings have significant implications for the development of treatment options for DOR and the expanded application of EA therapy.

DOR is characterised by a reduced number and quality of eggs within the ovary because of various factors. Clinical indications include decreased antral follicle count, elevated serum FSH levels, decreased E_2_ levels and compromised female fertility. If left untreated, DOR may progress to premature ovarian insufficiency (POI) or premature ovarian failure (POF). TG, derived from *Tripterygium wilfordii* and commonly employed as an immunosuppressant, has reproductive toxicity that can impair ovarian function, induce follicular atresia, disrupt endocrine balance and potentially induce DOR. In this study, rats in the DOR group exhibited disrupted oestrous cycles, characterised by prolonged metoestrus phases. Moreover, a reduction in the total number of oestrus days, a decrease in serum *E*_2_ levels, an increase in FSH levels, a significant decrease in the number of growing follicles at all levels and an increase in the number of AFs were observed. In addition, the CL exhibited a loose structure, with sparse distribution of capillaries, compared with that in the control group. These results suggest that TG administration resulted in a decline in ovarian reserve function in rats.

Inflammation is closely associated with ovarian function. Abnormal expression of inflammatory factors can lead to ovarian inflammation in situ, affect follicular development and ovulation [33] and lead to reduced ovarian reserves [34] and poor reproductive outcomes [35]. Inflammatory cell infiltration has been observed in the ovaries of rats with DOR [10]. In female reproductive tissues, including the ovaries, uterus, fallopian tubes and mammary glands, macrophages play a crucial role in regulating various processes, such as the pituitary-gonadal axis, ovarian cell proliferation, inflammation and steroid production [36]. Notably, a close association has been detected between M1 macrophages and ovarian senescence, as well as a decline in reserve function [37]. M1 macrophages possess the capacity to stimulate and activate primordial follicles (PRFs), hasten the depletion of primordial follicular reserves and accelerate ovarian senescence [18]. Conversely, the downregulation of M1 macrophages ameliorates ovarian dysfunction and diminishes the inflammatory response in autoimmune ovarian diseases [38]. In contrast, the polarisation of M2 macrophages diminishes the release of inflammatory mediators and exhibits therapeutic benefits in cases of POF [39]. Recent research findings have indicated that the introduction of M2 macrophage EV via injection can enhance the ovarian reserve and restore ovarian function in aged mice. The beneficial effect of M2 macrophage EV appears to be linked to the regulation of the ovarian inflammatory microenvironment. A recent single-cell sequencing analysis suggested that monocyte-derived macrophages associated with inflammation contribute to age-related ovarian dysfunction and diminished immune function [40]. These studies have shown that macrophage polarisation plays an important role in maintaining ovarian function.

The spleen is the largest secondary lymphoid organ in the human body and plays a crucial role in the immune system [41]. Upon migration from the spleen to the diseased tissue, macrophages release a significant amount of inflammatory factors and recruit neutrophils, thereby, driving inflammation and promoting tissue repair [42]. When macrophages are stimulated by inflammatory factors, such as IFN-*γ*, they polarise towards the M1 phenotype, characterised by the expression of surface markers, such as CD86, and the secretion of inflammatory factors, such as TNF-*α*. Conversely, when exposed to IL-4, they polarise towards the M2 phenotype, express markers, such as CD206, and secrete anti-inflammatory factors, such as IL-10 [43–47]. These two macrophage types possess antagonistic arginine metabolic pathways. iNOS, a characteristic enzyme of M1 macrophages, regulates the production of nitric oxide (NO) to mediate anti-infection and immunoinflammatory injury. In contrast, Arg1, a characteristic enzyme in M2 macrophages, hydrolyses arginine to regulate immune function, thereby, occupying the substrate of iNOS. This process downregulates NO production and facilitates tissue damage repair [48–51]. Our experimental results indicated that in the DOR group, the levels of pro-inflammatory cytokines (IFN-*γ* and TNF-*α*) increased, while those of anti-inflammatory cytokines (IL-4 and IL-10) decreased. Additionally, the protein and mRNA expression levels of iNOS increased, and those of Arg1 decreased, accompanied by an increase in CD86 expression. These findings suggest that TGs induce an inflammatory reaction and promote polarisation of M1 macrophages. This is consistent with the documented increase in serum IFN-*γ* and TNF-*α* levels in patients with POF, reduction in serum IL-4 levels in POF rats and increase in IFN-*γ* protein and mRNA expression in ovarian tissues [9, 52, 53]. These findings support the hypothesis that reproductive toxicity of TG stems from immunoinflammatory damage.

EA therapy is used in Chinese medicine as a form of treatment in which acupuncture is performed at specific acupoints while applying a biological current. It is commonly used to manage reproductive diseases [54, 55]. In our study, the ‘BL23', ‘CV12', and ‘CV4' acupoints were used for EA to treat DOR. According to the traditional Chinese medicine theory, proper functioning of the kidney and spleen–stomach systems is crucial for maintaining overall health. The Tiangui of kidney essence metaplasia, particularly related to female reproductive function, is closely associated with ‘BL23' on the back of the meridian theory. ‘BL23' is, therefore, utilised to nourish the kidney. Another key acupoint, ‘CV12' in the upper abdomen, is linked to stomach function, while ‘CV4' in the lower abdomen serves as a reservoir of vital energy in traditional Chinese medicine. In clinical practice, ‘CV4' is a frequently used acupoint for treating DOR, POI and perimenopausal syndrome [56] and a combination of these three acupoints is believed to enhance reproductive function. In this study, EA was found to ameliorate oestrous cycle irregularities in DOR rats, restore reproductive hormone levels to normal ranges, support normal follicular development, improve ovarian morphology and mitigate the deterioration of ovarian function in an animal model.

EA exerts notable anti-inflammatory and immunomodulatory effects. These effects are mediated through the regulation of immune cell levels, activation of the vagus nerve-adrenal axis, modulation of inflammatory factors and polarisation of macrophages. In the context of ischaemic stroke, EA has been shown to reduce small intestinal gamma–delta T cells, while increasing regulatory T cells, thereby, alleviating inflammatory damage [57]. Furthermore, EA applied to the sciatic nerve can modulate systemic inflammation by activating the vagus nerve via aromatic L-amino acid decarboxylase [58]. Additionally, low-intensity EA targeting the hindlimb regions is reported to activate the vagus nerve–adrenal axis, producing anti-inflammatory effects that depend on neuropeptide Y released from adrenal chromaffin cells [59]. Moreover, EA can enhance cardiac function in mice with myocardial infarction and assist in weight management in obese mice by regulating the expression of inflammatory factors and promoting the polarisation of M2 macrophages [26, 60]. Our previous research validated the ability of EA to modulate immune balance in DOR rats [27]. In this study, the effects of EA on macrophage polarisation and related inflammatory factors were examined to explore its mechanisms of action. Our results showed that through EA intervention, the levels of IFN-*γ* and TNF-*α* in the serum of DOR rats decreased, whereas those of IL-4 and IL-10 increased. Additionally, iNOS expression and the ratio of M1/M2 macrophages decreased, while Arg1 expression and the proportion of M2 macrophages increased. These results suggest that EA restores impaired ovarian function by facilitating the polarisation of macrophages to the M2 type, regulating immune balance and suppressing the immune inflammatory response in the ovaries.

The mechanism underlying the therapeutic effects of DOR has rarely been examined from the perspective of macrophages. Although it has been established that EA can influence macrophage polarisation, it is important to note that macrophages exhibit distinct polarisation tendencies at various stages of the inflammatory response. Therefore, the numerous regulatory pathways require further investigation. Our future research will focus on dynamic changes in macrophage polarisation in rats with DOR across various periods to identify the optimal timing for EA intervention. Additionally, we will further investigate the role of macrophage polarisation in DOR by utilising coculture techniques involving M1 and M2 macrophages, along with DOR ovarian tissues.

## 5. Conclusions

EA may prevent the decline in ovarian reserve function caused by TG. Moreover, safeguarding ovarian function may involve promoting the polarisation of macrophages towards the M2 type, as well as regulating the secretion of pro-inflammatory and anti-inflammatory cytokines to balance the M1/M2 macrophage ratio and inhibit the inflammatory response.

## Figures and Tables

**Table 1:** Primer sequences for quantitative reverse transcription-polymerase chain reaction (qRT-PCR) analyses.

Gene name	Primer sequences 5′→3′	Length (bp)
iNOS	Forward GGAGCGAGTTGTGGATTGT	146
	Reverse GAAGCCTCTTGTCTTTGACC	

Arg1	Forward AGACCACAGTATGGCAATTGGAAGC	107
	Reverse TTGTCAGCGGAGTGTTGATGTCAG	

GAPDH	Forward GACATGCCGCCTGGAGAAAC	92
	Reverse AGCCCAGGATGCCCTTTAGT	

## Data Availability

All data used and/or analysed in the current research are available from the corresponding author upon reasonable request.

## Nomenclature

DOR: Diminished ovarian reserve
TG: *Tripterygium* glycoside
TGs: *Tripterygium* glycoside suspension
EA: Electroacupuncture
FSH: Follicle-stimulating hormone
*E*_2_: Oestradiol
IFN-*γ*: Interferon-*γ*
TNF-*α*: Tumour necrosis factor-*α*
IL-4: Interleukin-4
IL-10: Interleukin-10
BL23: Shenshu
CV4: Guanyuan
CV12: Zhongwan
POI: Premature ovarian insufficiency
POF: Premature ovarian failure
EV: Extracellular vesicle.
